# Supplementary material for: Spatio-temporal visualization of light transport in complex photonic structures
Source: Light Sci Appl. 2016 May 6;5(5):e16090–. doi: 10.1038/lsa.2016.90 (PMC6059935; doi:10.1038/lsa.2016.90)
Supplement: Supplementary Information [file lsa201690x1.pdf]

Supplementary information: Spatio-temporal visualization of light transport in complex photonic structures

Lorenzo Pattelli*[1], Romolo Savo†[1], Matteo Burresi‡[2], and Diederik S. Wiersma[1,3]

[1]*European Laboratory for Non-linear Spectroscopy (LENS), Università di Firenze, 50019 Sesto Fiorentino (FI), Italy*
[2]*Istituto Nazionale di Ottica (CNR-INO), Largo Fermi 6, 50125 Firenze (FI), Italy*
[3]*Università di Firenze, Dipartimento di Fisica e Astronomia, 50019 Sesto Fiorentino (FI), Italy*

Probe and gate pulses characterization

A Spectra Physics Tsunami Ti:Sa laser is used to pump a Opal optical parametric oscillator, which provides a signal at (1510 ± 11) nm, an idler (not used in the experiment) and an unconverted residual at the same wavelength of the pump (i.e. (810 ± 5) nm). The average power of the signal and the residual beam is respectively 190 mW and 280 mW. Both pulses are characterized at the beginning of each measurement session spectrally and temporally. Figures S1a and b show typical spectra recorded for the Tsunami and the Opal, fitted with a $I(\omega) \propto \mathrm{sech}^2(\frac{\pi}{2}(\omega - \omega_c)\tau_p)$ function, as expected for an actively mode-locked Ti:Sa laser in a sub-ps configuration (i.e. featuring GDD compensation). Figure S1c shows an autocorrelation measurement of the residual pulse, fitted with the autoconvolution of two sech^2 pulses, which can be expressed as $I_{ac}(t) = \gamma\, \mathrm{cosech}^2(\gamma t)\,[\gamma \coth(\gamma t) - 1]$ with $\gamma = (2\log(1+\sqrt{2}))/(\tau_{fwhm})$. The full width half maximum duration of the original pulse is obtained by multiplication for the appropriate deconvolution factor, giving $\tau_{fwhm} = 0.6482 \times 144.6$ fs $= 93.7$ fs. To estimate the duration of the gate pulse we can rely on the cross-correlation measurement. Due to the

*Corresponding author
†Current affiliation: *Laboratoire Kastler Brossel, UMR 8552, CNRS, Ecole Normale Supérieure, Université Pierre et Marie Curie, Collège de France, 24 rue Lhomond, 75005 Paris, France*
‡Current position: *Gestione SILO Srl, via di Castelpulci 14/d, 50010 Scandicci (FI), Italy*

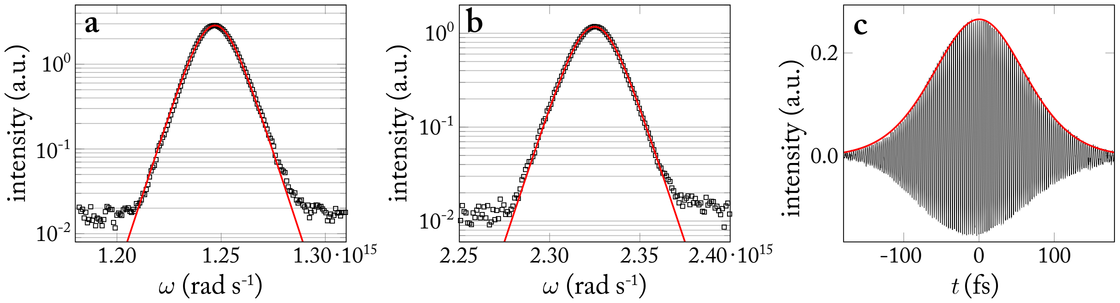

Figure S1: Pulses characterization. **(a)** Spectrum of the unconverted residual (probe pulse) from the OPO, with a $\mathrm{sech}^2(\omega)$ fit. **(b)** Spectrum of the OPO signal (gate pulse) with a $\mathrm{sech}^2(\omega)$ fit. **(c)** Autocorrelation measurement of the residual pulse. The envelope profile fringes has been fitted with the autoconvolution of two $\mathrm{sech}^2(t)$ pulses.

lack of any analytical expression for the cross-convolution of two different sech^2 pulses, we performed a fit plugging at each iteration the numerical convolution of the known pump pulse with an unknown sech^2 pulse (Fig. 1b). The routine eventually returned a full width duration of 134 fs for the gate pulse. The cross-correlation was measured with and without the lenses, to make sure that the optics used for the experiment were introducing negligible dispersion. The final full width half maximum, representing the instrument response function is of approximately 170 fs. The source term in all Monte Carlo simulation showed in this paper has been modeled as a sech^2 pulse of this duration, while its spatial distribution was set to a Gaussian beam with a waist of 10 µm, as given by our focusing lens.

Experimental setup details

The non-linear crystal used is a square 5 mm × 5 mm × 2 mm BBO crystal. Focal lengths are respectively 100 mm for L2 and L3, and 125 mm for the lens collecting the up-converted signal after the BBO. The final magnification obtained through the double imaging stages was roughly 1×. The focusing lens L1 is chosen so to provide a point-like excitation spot with respect to the typical length scales of the sample, which in our case was set to ∼ 10 µm. As far as the mentioned constraints on the wavevector distribution manipulation are satisfied, a different set of optics can be employed to obtain a different magnification. The CCD camera is a back-illuminated Andor iKon M912, with a 512×512 pixel sensor.

The optical elements collecting the diffusely transmitted intensity are placed at such a distance from the outer surface of the slab sample so to produce its focused image on the BBO crystal (at the probe beam wavelength). After the BBO, inside the shielded box, two optical paths can be alternatively selected. The first one is designed to work at the probe wavelength to ensure that the sample is correctly imaged in correspondence of the

BBO plane, and that the probe beam is correctly focused in correspondence of the rear surface of the sample. The second path is instead designed to work at signal wavelength and is used to correctly set the right focusing distance between the BBO and the CCD (which is different given the different wavelength).

BBO angular acceptance

Assuming narrow bandwidth pulses and a crystal cut at the proper critical angle for the wavelengths involved, the main source of k mismatch comes from misalignment of the k-vectors of the pump and gate photons. With reference to Figure 1d, assuming that the gate k-vector is aligned along the z axis, we can introduce a misalignment angle $\Delta\theta$ at which the conversion efficiency is halved. Its value can be estimated calculating

$$\frac{\mathrm{d}\Delta k}{\mathrm{d}\theta} = \frac{\mathrm{d}k_3}{\mathrm{d}\theta} - \frac{\mathrm{d}k_1}{\mathrm{d}\theta} - \frac{\mathrm{d}k_2}{\mathrm{d}\theta} = \frac{\omega_3}{c}\frac{\mathrm{d}n(\omega_3,\theta)}{\mathrm{d}\theta},$$

where k_1, k_2 and k_3 are respectively the pump, gate and signal k-vectors, and $\omega_3 = \omega_1 + \omega_2$ is the signal frequency. By plugging the expression that $n(\omega_3,\theta)$ must verify in a birefringent crystal as our BBO, we obtain

$$\frac{\mathrm{d}\Delta k}{\mathrm{d}\theta} = -\frac{1}{2}\frac{\omega_3}{c}n^3(\omega_3,\theta)\left[\frac{1}{n_\mathrm{e}^2(\omega_3)} - \frac{1}{n_\mathrm{o}^2(\omega_3)}\right]\sin 2\theta,$$

which might be regarded as the first term in a Taylor expansion around the correct angle $\hat{\theta}$. Considering that the halving efficiency condition requires $\sin^2(L\Delta k)/(L\Delta k)^2 = \frac{1}{2} \rightarrow L\Delta k \sim 2.783$ we finally obtain a full width half maximum angle $\Delta\theta$ of

$$\Delta\theta = \mp\left[L\frac{\pi}{2.783}\left(\frac{1}{\lambda_1} + \frac{1}{\lambda_2}\right)n^3(\omega_3,\hat{\theta})\left[\frac{1}{n_\mathrm{e}^2(\omega_3)} - \frac{1}{n_\mathrm{o}^2(\omega_3)}\right]\sin 2\hat{\theta}\right]^{-1} \simeq 0.1^\circ$$

for a ~ 2 mm thick β-barium borate crystal. This represents the absolute misalignment angle with respect to the extraordinary axis of the crystal (labeled as $\hat{z}$ in Fig. 1d) at which the conversion efficiency drops to 50 %. It is fundamental to note that, when projected over the xy or xz planes it takes respectively two very different misalignments $\Delta\theta_\mathrm{o}$ and $\Delta\theta_\mathrm{e}$ to reach this condition, which is the reason why the acceptance angle is weighted so differently along the two planar directions. Since we aimed at an isotropic conversion efficiency over the whole field of view, we introduced an iris (labeled as FF) in the Fourier plane of the collecting lens in order to have a low-pass isotropic k-vector filter prior to the up-conversion process. Based on the previous estimation this leads to an equivalent numerical aperture in air of some 10^{-3}. Different values can be obtained by varying the optical and physical properties of the crystal, such as its thickness. Depending on the specific application, different properties of the non-linear crystal should be considered. For example, Lithium borate (LBO) crystals exhibit a much larger angular acceptance

at the cost of a lower conversion efficiency, while Bismuth triborate (BiBO) offers both a better angular acceptance and a higher efficiency than a BBO and would therefore represent another ideal candidate for such imaging applications.

Polymer samples fabrication

Investigated polymer samples are made of a commercial UV-curing acrylate optical adhesive (Norland 65) with a dispersion of rutile nanoparticles with a diameter of 280 nm (Huntsman's Tioxide R-XL). The mixture of polymer and nanoparticles is rendered homogeneous through magnetic stirring and a ultrasonic bath ($\sim$ 1 h). We then let the resulting opaque paste be sucked by capillary forces inside an air gap of controlled thickness formed between two microscope glasses. The two glasses are firmly held apart by micro-spherical spacers of calibrated size. By spin coating in advance the microscope glasses with water-soluble polyvinyl alcohol we are able at a later stage to remove the glasses obtaining a flexible, free standing polymer slab. This notably allows to avoid multiple reflection of both pump and scattered light coming from the enclosing glasses.

Monte Carlo software

All simulations shown have been performed with a C++ software called MCPlus-Plus[1] based on the standard MCML Monte Carlo code for multi-layered slab samples[2]. Its source code is currently under development and is freely available at `http://www.lens.unifi.it/quantum-nanophotonics/mcplusplus/`. Benefiting from the object-oriented paradigm of C++, the software offers a high level of abstraction, scalability, modularity and ease of maintenance. Being completely written in C++, it can be executed an any hardware and can take extensive advantage of the multi-thread capabilities of most modern CPUs for increased performance. Other features worth mentioning include python scriptability and both raw and histogrammed output in the convenient H5 binary format.

Full 3D retrieval of ballistic speed of light

Spatio-temporal measurements of light traveling inside the grapefruit membrane and other analogue samples exhibit a transmitted intensity pattern which is determined by both ballistic and scattered light — where by "ballistic" we refer to light that undergoes roughly just two main scattering events: one to get inside the membrane and one to be scattered out. In particular, such ballistic component appears to persist for the whole time scale observed in our experiment and can be easily addressed by measuring the instantaneous position of the outer wavefront of the traveling pulse where, by definition, it is accumulated. This is confirmed by the steadily linear increase of the wavefront position. The retrieved slopes directly give the light speed along a certain direction inside

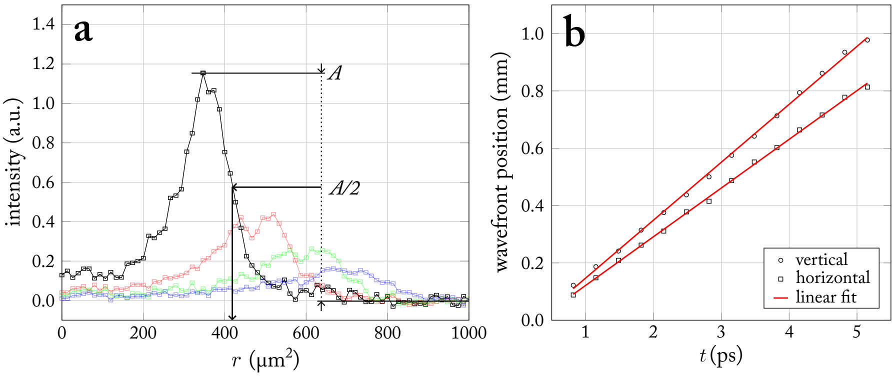

Figure S2: Grapefruit in-plane light speed retrieval. (a) For each measured profile, the instantaneous position of its outer wavefront is calculated at half its maximum amplitude A for both horizontal (shown) and vertical cross-cuts. **(b)** For the whole time window taken into account by our measurements the outer wavefront appears to travel ballistically with a well-defined speed. Linear fits return speeds of 170 µm ps^{-1} (0.57c) and 200 µm ps^{-1} (0.67c) for the horizontal and vertical axis respectively.

the slab specimen, which notably appears to be different along the x and y direction for the investigated sample. Combining these results with the perpendicular delay introduced by the intervening sample whose thickness is known (cfr. Figure 5a) we are able to retrieve the speed of light inside the membrane along each spatial dimension.

References

[1] Mazzamuto G, Pattelli L. *MCPlusPlus Documentation*. Florence: Italy, [updated 30 Jul 2015; cited 22 Sep 2015]. Available from: `http://www.lens.unifi.it/quantum-nanophotonics/mcplusplus/`

[2] Wang LH, Jacques SL, Zheng LQ. MCML-Monte Carlo modeling of light transport in multi-layered tissues. *Comput. Methods Programs Biomed* 1995; **47**: 131–146.
